# The genetic architecture of human skin pigmentation: evolution and adaptation across global populations

**DOI:** 10.3389/fgene.2026.1870791

**Published:** 2026-07-24

**Authors:** Arkopala Bose, Mainak Sengupta, Sumit Maitra, Arup Ratan Bandyopadhyay, Helmut Schaschl

**Affiliations:** 1 Department of Anthropology, Faculty of Science, University of Calcutta, Kolkata, India; 2 Department of Genetics, Faculty of Science, University of Calcutta, Kolkata, India; 3 Sociological Research Unit (SRU), Indian Statistical Institute, Kolkata, India; 4 Department of Evolutionary Anthropology, Faculty of Life Sciences, University of Vienna, Vienna, Austria

**Keywords:** adaptive trait, evolutionary hypotheses, gene-culture coevolution, polygenic architecture, population history, ultraviolet radiation

## Abstract

Human skin pigmentation is a dynamic and highly adaptive trait influenced by genetic, environmental, and cultural factors. It is driven by variation in ultraviolet radiation, governed by a complex polygenic structure, and further modulated through gene-culture interactions. This review synthesizes advances from evolutionary biology, anthropology, molecular genetics, and gene-culture coevolution to provide an integrated framework for understanding global diversity of pigmentation. We take a distinct approach by focusing on the major evolutionary hypotheses and the polygenic architecture underlying pigmentation. We emphasize both large-effect loci, including *SLC24A5*, *SLC45A2*, and *MC1R*, as well as population-specific adaptive variants identified through recent genomic and functional studies. Using evidence from diverse global populations, we examine how natural selection, demographic processes, and convergent evolution have jointly structured present-day variation in human skin pigmentation. We further emphasize how cultural practices, including clothing, diet, and mobility, have generated gene-culture feedback that modulates selective pressures over evolutionary timescales, underscoring the need for integrative, multi-ethnic research approaches that trace the temporal variation of skin pigmentation and advance its broader biomedical and anthropological implications.

## 1 Introduction

The evolution and global dispersal of anatomically modern humans (AMH) constitute one of the most consequential episodes of evolutionary history. Following their emergence in Africa approximately 300–200 thousand years ago (kya), *Homo sapiens* underwent a major expansion beyond the continent around 70–50 kya (Bergström et al., 2021), and subsequently spread across a wide range of environments, from equatorial tropics to high-latitude and high-altitude regions, exposing them to diverse regimes of solar ultraviolet radiation (UVR), temperature, and pathogen landscapes. These ecological contrasts, together with demographic processes such as bottlenecks, founder effects, and admixture, generated population-specific patterns of genetic variation through the combined action of natural selection and stochastic genetic drift, and contributed to the emergence of marked phenotypic diversity observed in present-day human populations (Parra, 2007; Sturm, 2009; Deng and Xu, 2018).

Among the most conspicuous manifestations of this diversity is human skin pigmentation. Variation in skin color is orchestrated by a complex polygenic architecture, involving population-specific alleles, intricate epistatic interactions, pleiotropic effects, and gene-specific evolutionary trajectories embodied by both global and regional selection pressures (Quillen et al., 2019). As a trait positioned at the interface between the organism and its environment, pigmentation has been shaped by spatially and temporally variable selective regimes, most prominently those associated with UVR, and also by broader ecological and physiological constraints. Besides, the evolutionary dynamics of pigmentation cannot be interpreted in purely genetic or environmental terms. Cultural innovations, including clothing, shelter, dietary practices, and mobility, have repeatedly modified patterns of UVR exposure, thereby modulating the intensity and direction of selection on pigmentation genes over time (Jablonski and Chaplin, 2000; Jablonski and Chaplin, 2010; Rocha, 2020; Jablonski, 2021).

Despite a vast literature on human skin pigmentation, most of the prior reviews have approached the topic in a fragmented manner, often emphasizing either molecular mechanisms, specific evolutionary hypotheses (particularly those centred on UVR, vitamin D, and folate), or health-related consequences in isolation. While these perspectives have been essential for establishing the selective context in which pigmentation evolved, they tend to obscure a broader and increasingly evident insight: global pigmentation diversity cannot be explained by a single environmental gradient or a single evolutionary mechanism. Instead, it reflects a long-term interplay among genetic architecture, demographic patterns, ecological context, and cultural buffering, unfolding over deep evolutionary time.

A few of the previous reviews have substantially synthesized and advanced the understanding of the evolutionary biology of human skin pigmentation. Collectively, these studies emphasized the evolutionary drivers of skin pigmentation, including vitamin D-folate trade-offs, thermoregulatory adaptations, and the interplay among UVR, physiology, and cultural practices (Jablonski, 2021). Some studies synthesized evidence on the molecular genetics and evolutionary history of pigmentation loci, highlighting major genes and patterns of natural selection across populations (Sturm, 2009; Quillen et al., 2019). Building upon these foundations, we present an integrative synthesis that expands the current evolutionary frameworks by examining human skin pigmentation as a trait evolving within a multidimensional selective landscape, governed by genetic variation, and further shaped by cultural modulation that restructures exposure and shifts selective optima. Within this framework, we examine how distinct genetic architectures have responded to region-specific adaptive regimes. Drawing on recent advances in multi-ethnic genome-wide association studies (GWAS), functional genomics, ancient DNA research, and gene-culture coevolution theory, this review aims to clarify how molecular pathways, population history, and cultural practices have jointly structured one of the most dynamic dimensions of human adaptation.

## 2 Evolutionary and genetic basis of human skin pigmentation

### 2.1 Evolutionary origin and adaptive significance

The evolution of skin pigmentation in the genus *Homo* represents a striking yet underrepresented example of human adaptation. Compared with other cardinal traits such as encephalization, bipedal locomotion, or technological innovation, pigmentation has traditionally been treated more as a descriptive taxonomic character rather than a functional adaptive trait. Early anthropological classifications often relied on skin color as one of the criteria for broader population categorization (Jablonski, 2020). From the ecological observations of Gloger (1833) (Gloger, 1833), to recent advances in paleontology, archaeology, genomics, and environmental physiology now highlight skin pigmentation as a key adaptive interface, linking human biology to diverse ecological and cultural landscapes (Rocha, 2020; Jablonski, 2021; Pavan and Sturm, 2019).

More broadly, skin pigmentation is a pivotal biological trait that shows both intra- and interspecies heterogeneity and serves diverse adaptive functions. Comparative analyses across species reveal the multifaceted pathways organisms adopt to modulate pigmentation according to ecological circumstances: to avoid predators, thermoregulate, communicate with conspecifics, camouflage, and protect against UV (Liu et al., 2024). Different species have developed diverse survival strategies to thrive in their ecological niche. For example, cephalopods have chromatophores, whereas reptiles and amphibians have melanophores and iridophores to produce dynamic color patterns and physiological color changes that foster camouflage and thermoregulation. Aves have xanthophores that aid in structural coloration, rendering vibrant plumage. Mammals have melanophores that produce pale to dark pelage as a means of camouflage and thermoregulation (McNamara et al., 2021). However, human skin coloration by contrast, reflects a unique adaptive process, shaped by long-term genetic, ecological, and cultural pressures rather than rapid physiological color changes (Jablonski, 2021).

Reconstructing the ancestral condition of skin pigmentation is crucial for understanding its evolutionary trajectory. Early hominins likely had integument similar to that of modern chimpanzees: lightly pigmented or unpigmented skin covered by a dense coat of dark hair, with exposure limited to regions such as the face, ears, palms, and soles, as inferred from existing primate data (Post et al., 1975). This ancestral “plumage” was gradually lost, and it facilitated the development of an efficient thermoregulatory system based on whole-body eccrine sweating, allowing sustained physical activity in hot, humid, and open environments. Coupled with striding bipedalism, these changes increased metabolic heat production and likely intensified selective pressure for both hair loss and the expansion of eccrine sweat glands. This evolutionary shift appears to have occurred simultaneously with the emergence of skin pigmentation, forming an integrated thermoregulatory and physiological complex that supported prolonged endurance, heat dissipation, and resilience to diverse environments (Jablonski and Chaplin, 2010; Jablonski, 2004).

Fossil evidence supports the timing and anatomical basis of these transitions. *Ardipithecus ramidus*, dated ∼4.4 million years ago (Mya), and *Australopithecus afarensis* (∼3.2 Mya) exhibit traits consistent with habitual bipedalism and woodland-savanna foraging (Gibbons, 2009; Johanson et al., 1978). Around ∼1.8 Mya, *Homo erectus*, as exemplified by the Nariokotome Boy, had evolved longer limbs, modern body proportions, and enhanced mobility, enabling long-distance travel, tool use, and cooperative hunting (Brown et al., 1985). These activities elevated brain temperature, challenging cerebral homeostasis, and likely contributed to the selective advantage of thermoregulatory adaptations in naked skin, which may have contributed to the expansion of brain size in early *Homo* (Wheeler, 1985; Nelson and Nunneley, 1998).

However, naked skin also exposed hominins to intense solar UVR, which can cause DNA damage, generate reactive oxygen species (ROS), and deplete folate, an essential nutrient for fetal development and cell division (Williams et al., 2015). These deleterious effects imposed selective pressure for photoprotection, and the evolutionary response was increased melanization of the skin. Eumelanin, the dominant pigment found in dark skin, absorbs UVR effectively and neutralizes ROS, thereby protecting the underlying tissues from oxidative and mutagenic damage (Nielsen et al., 2006). Over time, the development of melanin-rich skin became a vital adaptation that allowed hominins to thrive in sun-exposed environments (Rogers et al., 2004).

### 2.2 Selective pressures underlying the evolution of human skin pigmentation

**FIGURE 1:**
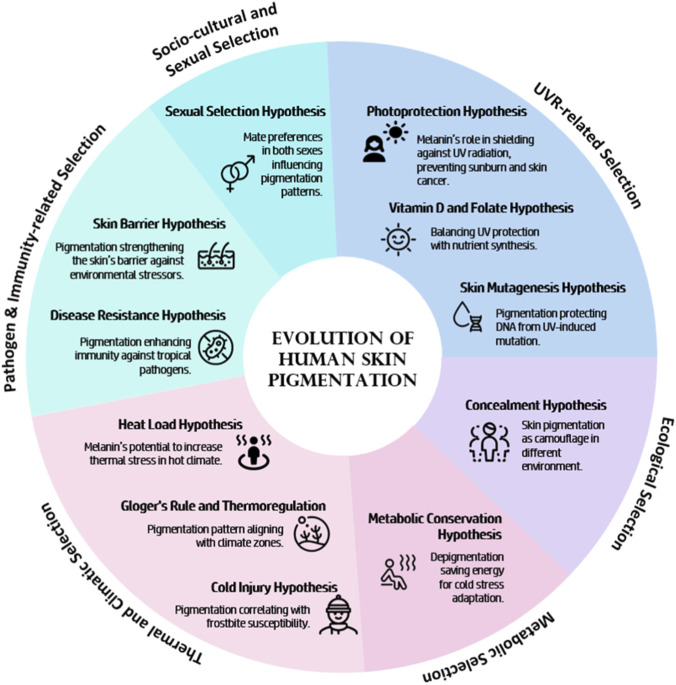
Major evolutionary hypotheses and selective forces explaining human skin pigmentation diversity. This circular infographic illustrates major adaptive hypotheses proposed, at different times and from different disciplinary perspectives, to explain the evolution of human skin pigmentation across diverse populations. Together, these hypotheses synthesize the multifactorial and context-driven evolution of pigmentation in *Homo sapiens*.

The origin and physiological dynamics of human skin pigmentation provide the framework for understanding its adaptive diversity. Human skin pigmentation is not a simple Mendelian trait, but a highly polygenic phenotype shaped by long-term interactions among genes, environment, and culture, which can be understood through multiple selective pressures. Its global variation reflects both directional and stabilizing selection operating under different ecological and demographic contexts. Multiple hypotheses have been proposed (Figure 1), encompassing ultraviolet radiation-driven to ecological, metabolic, climatic, pathogenic, and sociocultural influences, that together articulate distinct yet intersecting selective pressures collectively shaping the adaptive diversification of human skin pigmentation.

#### 2.2.1 UVR-driven selection

Photoprotection hypothesis: Some of the earliest explanations for skin pigmentation focused on the role of melanin in protecting the skin from harmful UVR (Pathak et al., 1976). In equatorial regions, dark skin clearly minimizes UV-induced damage like sunburn and skin cancer (Jablonski and Chaplin, 2000). Yet Blum (1961) argued that most skin cancers develop after reproductive age and rarely cause death, making it unlikely that skin cancer prevention itself was a major selective force. He instead suggested that severe sunburns, by impairing foraging and hunting efficiency, may have had more fitness consequences, making photoprotection a more relevant survival benefit (Blum, 1961). However, the existence of prolonged post-reproductive lifespans and grandparental investment in humans can contribute substantially to inclusive fitness (Hawkes et al., 1998; Davison and Gurven, 2022), indicating that the evolutionary relevance of late-life UV-induced damage may not be negligible.

Vitamin D_3_ and folate hypothesis: This hypothesis explains the pigmentation diversity as an evolutionary trade-off shaped by UVR. In this view, skin pigmentation balances photoprotection against the need for efficient nutrient synthesis. In low UVR regions such as much of northern Europe, depigmentation enhances cutaneous vitamin D_3_ production, which is essential for skeletal health, immune response, reproductive health, and several other physiological functions (Murray, 1934; Loomis, 1967). Meanwhile, in tropical and equatorial regions, dark pigmentation protects against UV-induced photolysis of folate, a micronutrient crucial for embryonic viability and fetal development (Branda and Eaton, 1978; Yafei et al., 2012). The interaction between these opposing selective pressures is presumed to underlie the latitudinal clines of pigmentation, reflecting an adaptive compromise rather than a unidirectional response to UVR (Jablonski and Chaplin, 2000; Jablonski and Chaplin, 2010; Jablonski, 2004).

Skin mutagenesis hypothesis: The Skin Mutagenesis Hypothesis emphasizes the role of pigmentation in limiting UVR-induced DNA damage and mutagenesis (Greaves, 2014). By preserving genomic integrity, pigmentation indirectly supports DNA repair processes and cellular function involving vitamin D_3_ and folate (Williams et al., 2015; Mason et al., 2010). While this provides a plausible mechanistic account for the protective function of pigmentation, its direct evolutionary importance remains debated. Most skin cancers develop late in life, thereby contributing little to differences in reproductive success (Jablonski and Chaplin, 2014). Recent studies on fair-skinned populations living in high-UV environments, such as Australia, show that, although metastatic skin cancers are common among them, their onset overwhelmingly occurs post-reproductive, making it difficult to consider skin cancer itself as a direct selective force in early hominin evolution (Okholm et al., 2026). Nonetheless, the distinction between the reproductive and post-reproductive fitness may not be absolute in humans, suggesting late-onset traits could still exert indirect evolutionary consequences (Hawkes et al., 1998; Davison and Gurven, 2022).

#### 2.2.2 Ecological selection

Concealment Hypothesis: Concerning ecological contexts, the Concealment Hypothesis suggests that skin pigmentation may have functioned as camouflage, with darker pigmentation facilitating concealment in low-light forested environments (Cowles, 1959). However, this explanation becomes less convincing when considered across the wide range of habitats hominins have occupied. In open grassland habitats, dark pigmentation would likely have increased visual conspicuousness, and although light skin may offer camouflage in snowy northern regions, such effects would be limited in heterogeneous or seasonally variable landscapes. Concealment, therefore, alone is insufficient to account for the global diversity of human skin pigmentation.

#### 2.2.3 Metabolic selection

Metabolic conservation hypothesis: Building upon metabolic trade-offs, the metabolic conservation hypothesis suggests that depigmentation in certain populations may have been favored not only to improve vitamin D_3_ synthesis under low UVR but also to save metabolic energy otherwise allocated to melanin production. The energy saved this way would have been redirected toward other physiological demands, such as adaptation to cold stress and fluctuating resource availability (Elias and Williams, 2016; Elias and Williams, 2013). Within this framework, pigmentation variation is seen as part of metabolic selection, rather than UV exposure alone.

#### 2.2.4 Thermal and climatic selection

Heat Load Hypothesis: While photoprotection focuses on UV effects, the Heat Load Hypothesis emphasizes the role of climate and temperature rather than UVR alone (Blum, 1961). It argues that because melanin absorbs heat, dark skin might increase thermal stress in hot deserts and cause dehydration. However, this argument has been challenged on ecological and geological grounds, as early hominids evolved primarily in humid savannahs, not extreme deserts, and the Sahara’s current hyper-arid conditions are geologically recent (Boos and Korty, 2016), implying that heat load alone cannot explain pigmentation.

Gloger’s rule and thermoregulation hypothesis: This temperature-based explanation is closely related to Gloger’s Rule (1833), which notes many animal species tend to have darker pigmentation in humid tropics and lighter pigmentation in temperate, drier zones (Gloger, 1833; Delhey, 2019). However, this argument faces a paradox: dark pigmentation absorbs more heat, yet is common in hot regions. Rensch (1929) later refined this observation, suggesting pigmentation strongly correlates with humidity, rather than with temperature, explaining regional skin pigmentation patterns more comprehensively (Delhey, 2019).

Cold injury hypothesis: Complementing disease-related arguments, this hypothesis (Post et al., 1975) suggests that melanocytes may be particularly vulnerable to freezing, making dark skin more susceptible to frost injury (Steegmann, 1967). This may help explain why heavily pigmented skin is rare in cold climates, introducing temperature as a factor beyond UV or pathogens (Roberts and Kahlon, 1976). But this, too, is best considered as a contributing constraint rather than a primary driver.

#### 2.2.5 Pathogen and immunity-related selection

Skin Barrier Hypothesis: Recognizing the complex environmental challenges faced by early humans, the Skin Barrier Hypothesis proposes that darker skin pigmentation evolved in part to strengthen the skin’s barrier function against UVR, heat, and aridity, helping to reduce water loss and maintain epidermal integrity (Elias et al., 2009). This hypothesis offers a physiological complement to the photoprotection and thermoregulation explanations of pigmentation evolution. While this perspective usually broadens the functional context of pigmentation, substantial population-level variation in skin morphology and barrier properties complicates its use as a general explanation (Elias and Williams, 2016).

Disease Resistance Hypothesis: Other authors have argued more directly for a role of disease in pigmentation. Wassermann’s Disease Resistance Hypothesis reflects that pigmentation evolved to enhance immunity against tropical pathogens rather than primarily to regulate UV exposure (Wassermann, 1965). He proposes links between pigmentation, reduced adrenocortical activity, stimulating immune defenses, and increasing melanin via hormonal pathways. Mackintosh later reinforced this aspect of the hypothesis by emphasizing antimicrobial properties (Mackintosh, 2001). Although this perspective offers an immunological function to pigmentation beyond UV protection, its explanatory power is limited by the relatively recent emergence of many major infectious diseases, such as malaria, following the advent of agriculture approximately 6 kya (Joy et al., 2003).

#### 2.2.6 Sociocultural and sexual selection

Sexual selection hypothesis: Moving beyond purely environmental explanations, this hypothesis proposes that social and mating preferences may have contributed to variation in human skin pigmentation. Darwin, and later Aoki (2002), suggested that assortative mating or sex-biased preferences could have favored lighter-skin tones in certain populations. Humans exhibit a consistent sexual dimorphism, with females generally having less pigmented skin than males within the same population, a pattern thought to be influenced by hormonal regulation (van den Berghe and Frost, 1986). In societies where female-biased mate choice, coupled with intense male-male competition, is prevalent, sexual selection may favor pigmentation in both sexes over time. Conversely, in societies where males have most control over mate choice, and a surplus of females compete for mates, sexual selection may favor depigmentation in both sexes over time (Frost, 1994). However, evidence for such preferences appears to be culturally and temporally inconsistent, rather than universal. Sexual selection is therefore best regarded as a secondary or modifying force. It interacts with ecological pressure and social structure to shape pigmentation patterns in specific populations (Madrigal and Kelly, 2007).

Rather than representing mutually exclusive explanations, these processes operate at different causal levels: UV-driven selection defines the primary selective axis, barrier and immune functions modulate local optima, and sociocultural factors dynamically reshape exposure regimes. Together, they define a multi-layered adaptive landscape rather than a single-factor model, for the diverse evolutionary trajectories and genetic architectures that underlie the global diversity of skin pigmentation.

### 2.3 The genetic architecture of human skin pigmentation

**FIGURE 2:**
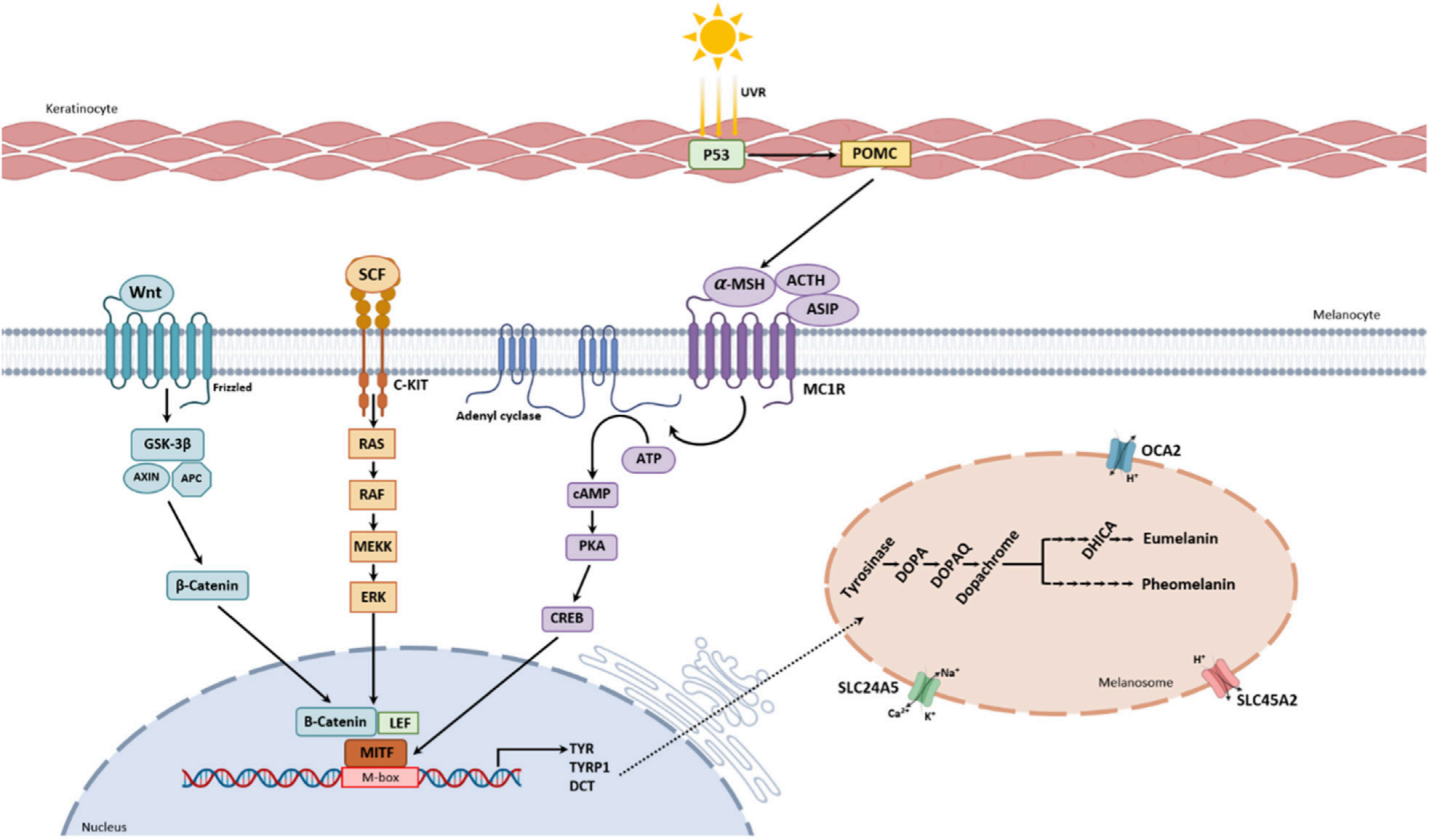
Molecular signaling pathways regulating melanogenesis in human skin. This schematic illustrates the core signaling pathways that regulate melanocyte activity and melanosome biogenesis. It highlights the role of key molecular mediators, including stimulatory and inhibitory factors, and illustrates their regulations in response to internal and environmental cues. Created with BioRender.com.

The investigation of the genetic architecture of human skin pigmentation has advanced significantly over the past 2 decades. What began as early gene discovery and identification of candidate genes has transitioned into increasingly comprehensive models of polygenic variation, modulated by regulatory and environmental factors. Furthermore, recent advances in genomic and functional studies extend this knowledge and have substantially elucidated the evolutionary trajectory of skin pigmentation in different populations. In this section, we briefly summarize the molecular basis of melanogenesis and then place it in the context of current understanding of genetic architecture and evolutionary dynamics derived from recent genomic and functional studies (Figure 2).

#### 2.3.1 From candidate genes to polygenic models

Foundational studies, such as those by Sturm and colleagues (Sturm, 2009; Sturm and Duffy, 2012), identified key pigmentation genes (e.g., *MC1R*, *OCA2*, *TYR*) and demonstrated the evolutionary and functional consequences of their polymorphisms. Skin pigmentation is now recognized as a trait influenced by a combination of large-effect loci (e.g., *SLC24A5*, *SLC45A2*) and a multitude of small-effect variants distributed across the genome, and embedded in complex regulatory networks with gene-environment interplay further shaping the phenotypic variation. This understanding has been further transformed by leveraging ancient DNA (aDNA) to trace the evolutionary dynamics of pigmentation variants (Perretti et al., 2025; Ferrando-Bernal et al., 2025). Together, functional and comparative genomic studies indicate that the melanogenic pathway constitutes a conserved biochemical core onto which different populations have layered distinct genetic architectures, producing similar or divergent phenotypes through different evolutionary routes.

#### 2.3.2 Molecular pathways underlying pigmentation

The core biochemical process of pigmentation is melanogenesis within melanocytes, governed by a tightly regulated mechanism involving synergistic interactions between various genes, signaling pathways, cellular organelles, and environmental factors. This enzymatic cascade is initiated by Tyrosinase (*TYR*) and its associated genes tyrosinase-related protein 1 (*TYRP1*) and dopachrome tautomerase (*DCT*), which catalyze the rate-limiting steps of melanin synthesis. This pathway is transcriptionally controlled by microphthalmia-associated transcription factor (*MITF*), the master regulator of melanogenesis. *MITF* activity is primarily regulated through the α-MSH-MC1R-cAMP axis, in which *MC1R* signaling activates protein kinase A (PKA)-dependent phosphorylation of CREB, thereby inducing *MITF* expression. *MITF* enhances the melanogenic activity of *TYR*, *TYRP1*, *DCT*, and regulates melanocyte survival and differentiation, coordinated by the SCF-cKIT signaling through the MAPK/ERK cascade and the WNT/β-catenin axis (Herraiz et al., 2021). Beyond transcriptional control, multiple transporter and ion-exchange protein coding genes, including *OCA2*, *SLC24A5*, *SLC45A2*, and *MFSD12*, regulate melanosomal pH, and enzyme trafficking, thereby exerting strong quantitative effects on pigmentation (Pavan and Sturm, 2019; Feng et al., 2024). Melanosome biogenesis and maturation further depend on lysosomal trafficking genes, such as *BLOC-1* complex genes, as well as the endosomal recycling machinery like *VPS39*, *COMMD3* (Bajpai et al., 2023). Mature melanosomes are then transported along microtubules into keratinocytes, where they form supranuclear caps that protect genomic DNA from UV-induced damage. The UVR exposure further modulates melanogenesis through p53-mediated upregulation of *POMC* and *KITLG* in keratinocytes, increasing α-MSH production and stimulating melanogenesis. In contrast, antagonists such as agouti signaling protein (ASIP) and modulators such as β-defensin 3 (HBD3) fine-tune this regulatory network by attenuating *MC1R* activation (Pavan and Sturm, 2019).

#### 2.3.3 Technological advances and contemporary approaches

Recent methodological innovations have significantly advanced the mapping and functional characterization of pigmentation-associated loci. Large-scale GWAS (Stokowski et al., 2007; Crawford et al., 2017; Visconti et al., 2018; Jonnalagadda et al., 2019; Adhikari et al., 2019; Wang et al., 2022; Seo et al., 2022; Kim et al., 2024) across diverse populations identified numerous novel loci contributing to pigmentation variation. Functional genomic approaches such as massively parallel reporter assays (MPRA), hi-C chromatin interaction mapping, and CRISPR-based functional screens have further demonstrated that many of these signals map to enhancer-promoter and causative non-coding variants rather than to protein-coding changes, revealing a dense regulatory architecture underlying pigment biology (Feng et al., 2024; Bajpai et al., 2023). More recently, machine learning and artificial intelligence approaches applied to genomic data analyses have begun to identify previously unrecognized loci and complex interaction networks, and to predict pigmentation phenotypes with higher accuracy (Tripathi et al., 2024). Looking forward, the integration of population-scale genomics with computational approaches and multi-omics data, including epigenomics, transcriptomics, and proteomics, is expected to enable an increasingly robust reconstruction of the evolutionary history and adaptive dynamics of human skin pigmentation, unifying molecular genetics, evolutionary biology, and human diversity within an integrative framework.

### 2.4 Adaptive signatures and global polymorphic diversity of human skin pigmentation genes

**FIGURE 3:**
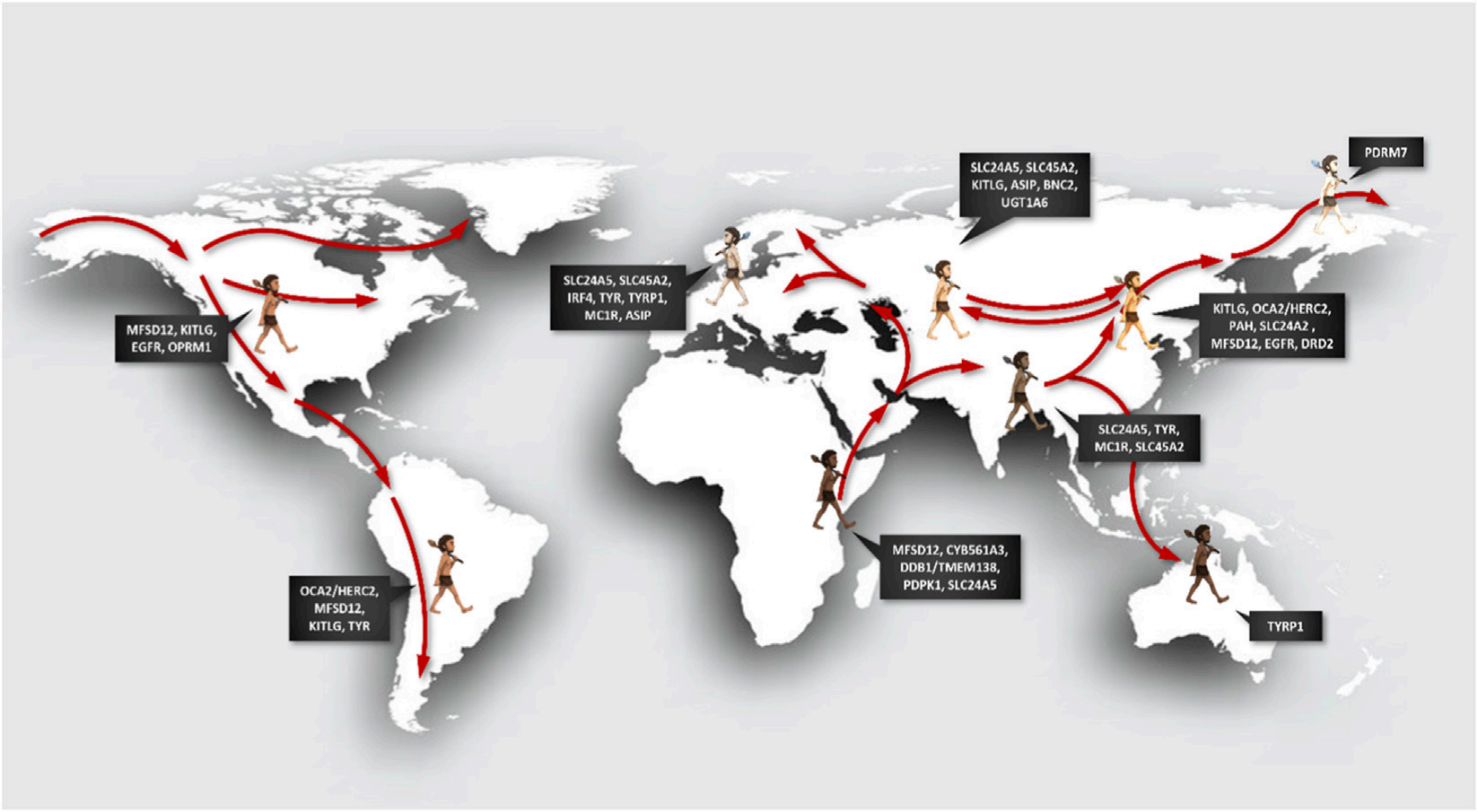
Global human migration routes and region-specific pigmentation-associated genetic variants. This map illustrates major migratory trajectories of anatomically modern humans across continents, alongside the geographic distribution of key genetic loci implicated in pigmentation traits. As populations dispersed from Africa and encountered diverse UV radiation regimes, selective pressures shaped the prevalence of alleles in genes such as SLC24A5, SLC45A2, MFSD12, OCA2/HERC2, TYR, TYRP1, MC1R, and KITLG**,** shaping regional patterns of skin pigmentation.

Across human evolutionary history, skin pigmentation has been shaped by a complex interplay of natural selection, migration, genetic drift, and demographic history. Variation in UVR across geographic regions has been a major selective pressure, influencing the distribution and frequency of pigmentation-associated alleles in global populations. Recent advances in functional genomic studies and GWAS have revealed that human skin pigmentation is orchestrated by a polygenic structure involving both large-effect loci (e.g., *SLC24A5*, *SLC45A2*, *MC1R*) and numerous small-effect variants distributed across the genome, as discussed in Section 2.3. However, understanding these molecular mechanisms alone cannot fully explain the striking diversity of skin pigmentation observed globally. Ongoing genomic studies have deciphered the polymorphisms in various pigmentation genes, mostly with a clear signature of positive selection in specific regions (Figure 3).

**FIGURE 4:**
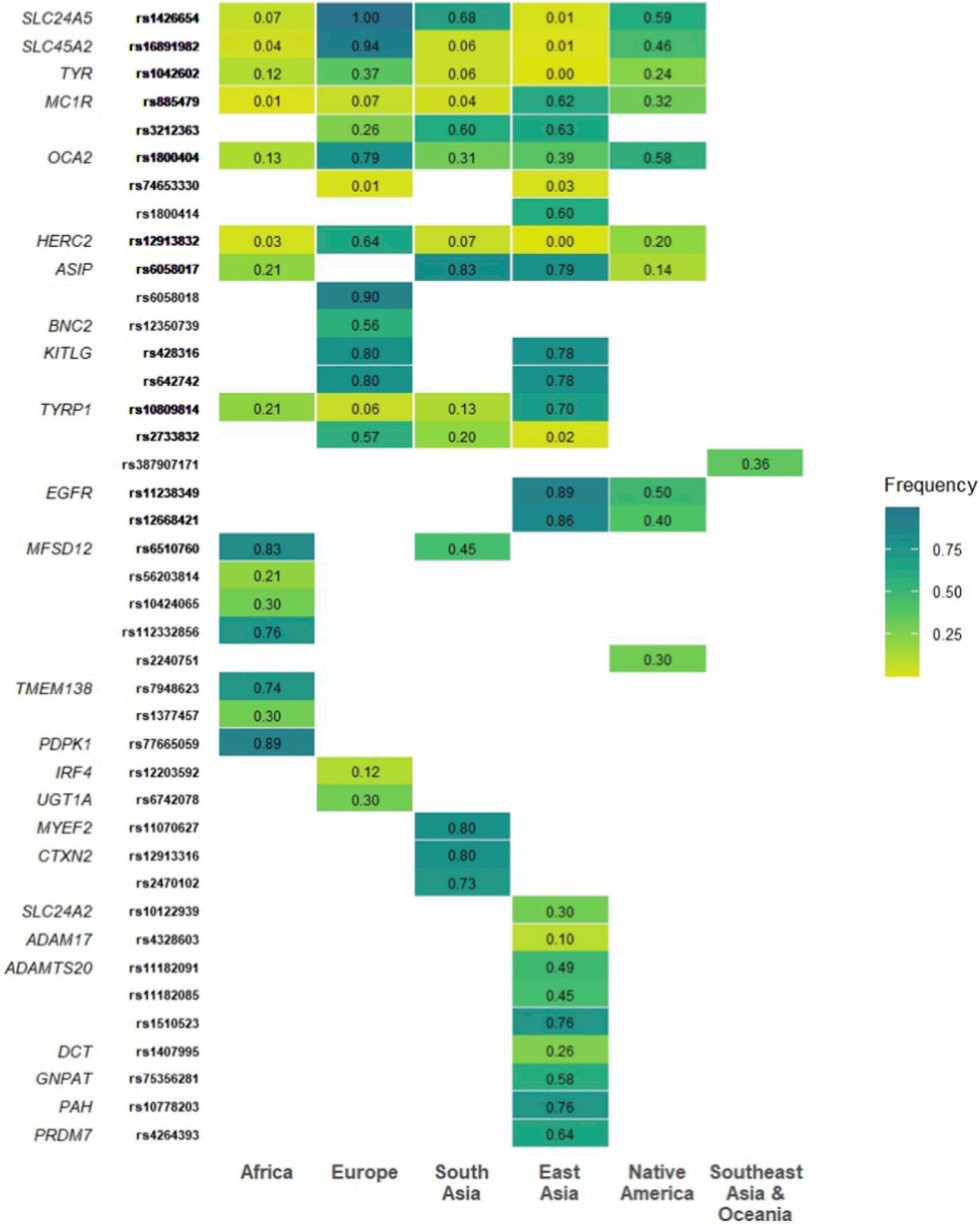
Global distribution of pigmentation-associated variants across populations. Heatmap showing derived allele frequency distribution of the region-specific pigmentation-associated variants across six major geographical regions. Allele frequency data were primarily obtained from the 1,000 Genome Project Phase 3 dataset (Auton et al., 2015). For a subset of variants (*MFSD12* rs2240751, *GNPAT* rs75356281, *PRDM7* rs4264393) lacking comprehensive representation in this dataset and showing population-specific enrichment, allele frequencies were derived from published literature sources (Adhikari et al., 2019; Yang et al., 2022; Malyarchuk, 2022) respectively.

The geospatial distribution and frequency of these genetic variants vary tremendously from highly melanized skin in equatorial African populations to depigmented skin in northern Europe, with broad clinal variation observed across Asia and the Americas. To illustrate these global patterns, we present a comparative heatmap of derived allele frequencies across six major geographic regions (Figure 4), primarily based on data from the 1000 Genomes Project Phase 3 dataset (Auton et al., 2015). For several population-specific variants that are not comprehensively represented in this dataset, allele frequencies were derived from published sources (Adhikari et al., 2019; Yang et al., 2022; Malyarchuk, 2022). The limited availability of comparable allele-frequency data for several loci, particularly in Southeast Asian and Oceanian populations, reflects their broader underrepresentation in global genomic data resources. This underrepresentation continues to hinder a comprehensive reconstruction of the evolutionary history of human pigmentation beyond the best-studied major continental populations.

The regional patterns explicated below therefore represent distinct ways in which the underlying evolutionary processes operate on a shared melanogenic system, under diverse ecological and demographic contexts.

#### 2.4.1 Africa

The evolutionary gestation of skin pigmentation originated in Africa, where strong and long-term selection has favoured dark skin since it protects against high and persistent UVR, thereby reducing folate photolysis and DNA damage (Jablonski, 2021). However, there exists substantial regional diversity, ranging from the extremely high melanin levels observed among equatorial Nilo-Saharan pastoralists in East Africa to the comparatively lighter skin of San hunter-gatherers in southern Africa (Crawford et al., 2017).

Genomic studies suggest that, although the UVR gradient remains the primary selective force shaping the African pigmentation patterns, the observed diversity reflects signals associated with multiple loci involved in melanogenesis and its regulation. These loci include *MFSD12*, *DDB1/TMEM138*, *OCA2/HERC2*, *HMG20B*, *MITF*, *LEF1*, *TRPS1*, *BLOC1S6*, *CYB561A3, PDPK1*, and *SLC24A5* (Feng et al., 2024; Crawford et al., 2017; Fan et al., 2023). Among these, *MFSD12* provides a clear example of how regulatory variation can influence pigmentation. In East African populations, particularly Nilo-Saharan groups, reduced expression of *MFSD12* is associated with higher melanin levels, and the presence of its ancestral alleles at several associated regulatory SNPs (rs56203814, rs10424065, rs6510760, rs112332856) is consistently associated with darker pigmentation (Crawford et al., 2017).

A second important cis-regulatory region encompasses the *DDB1* and its neighboring gene *TMEM138*, which are involved in responses to UV-induced DNA damage. Several variants in this region, including rs7948623, rs1377457, and rs148172827 show strong associations with darker skin in East Africa (Feng et al., 2024). A functional study has demonstrated that rs7948623 variant acts as an enhancer for *CYB561A3* gene, which encodes a melanosomal protein. Altered expression of this protein affects multiple downstream pigmentation genes (Feng et al., 2024). The distribution of these alleles, including their enrichment in populations with high-UV exposure and the near-fixation of alternative alleles in lighter-skinned Eurasian populations, highlights the importance of regionally structured selection acting through regulatory mechanisms.

In contrast, the relatively lighter pigmentation observed in some San and KhoeSan populations reflect a unique demographic and evolutionary history. In southern African KhoeSan populations, the derived light pigmentation-associated *SLC24A5* rs1426654-A allele was likely introduced through East African pastoralist-related gene flow within the last ∼2 kya and subsequently rose to high frequency, consistent with strong positive selection (Lin et al., 2018). In addition, whole-genome sequencing identified the ancestral rs77665059-C variant within a melanocyte-active enhancer of *PDPK1*; this allele is enriched in San populations, associated with lighter pigmentation, and may influence skin color by modulating PDPK1 enhancer activity and gene expression.

Taken together, these findings show that African pigmentation diversity cannot be explained by a single continental model of UVR adaptation. Instead, it reflects regionally structured selection acting on multiple pigmentation and regulatory pathways. In equatorial and high-UV regions, darker pigmentation is associated with ancestral or regulatory variants that increase melanin production and enhance photoprotection, whereas the comparatively lighter pigmentation observed in some southern African San and KhoeSan populations reflects a distinct combination of recent adaptive introgression at *SLC24A5*, regulatory variation involving *PDPK1*, and recent selection on radiation-response pathways (Fan et al., 2023; Lin et al., 2018; Skoglund et al., 2017). In the case of *SLC24A5*, reduced UVR exposure and possible Holocene dietary shifts associated with pastoralism have been proposed as selective pressures, although the precise biological or cultural drivers remain unresolved (Lin et al., 2018). Thus, African pigmentation evolution has been shaped by fine-scale regional demographic histories, gene flow, local adaptation, and UVR-mediated selection.

#### 2.4.2 Europe

In contrast to Africa, the evolution of light skin pigmentation in Europe is distinguished by a genetic architecture dominated by a small number of large-effect loci that underwent recent, strong directional selection (Jablonski, 2021). Although AMH entered Europe by at least ∼43 kya, most aDNA studies indicate that Upper Paleolithic and many Mesolithic West Eurasian individuals carried relatively few light skin pigmentation-associated alleles that are common in present-day Europeans (Olalde et al., 2014). This suggests that the pigmentation profile characteristic of contemporary Europeans emerged gradually and relatively recently, primarily between ∼11–5 kya through a combination of migration, admixture, and post-admixture selection (Ju and Mathieson, 2021). A significant contribution came from Neolithic farmer populations of Anatolian ancestry, who introduced the derived light pigmentation-associated *SLC24A5* rs1426654-A allele (p.Ala111Thr) into Europe during their northward and westward expansion. This allele rose rapidly in frequency in Early Neolithic Europe, largely through migration, and continued to be subject to selection after admixture with local hunter-gatherer populations (Mathieson et al., 2015). In contrast, light pigmentation-associated variation at *SLC45A2*, particularly rs16891982 (p.Leu374Phe), was initially present at lower frequency in ancient European populations but underwent strong positive selection during the Holocene, eventually approaching very high frequency in many present-day Europeans (Mathieson et al., 2015; Irving-Pease et al., 2024). Recent large-scale aDNA analyses further support strong selection at the *SLC45A2* locus, with selected haplotypes increasing in frequency from the late Pleistocene/early Holocene and plateauing at high frequency by the late Holocene (Irving-Pease et al., 2024). Additional pigmentation-associated loci, including *HERC2/OCA2*, *TYR* and *GRM5*, also show evidence of allele-frequency change or selection during this period, reflecting the complex evolution of skin, hair, and eye pigmentation in European populations (Mathieson et al., 2015; Irving-Pease et al., 2024). Collectively, these findings indicate that the pigmentation-associated allele combination characteristic of many present-day Europeans is a relatively recent outcome of Holocene migration, admixture, and strong selection, reaching its current high frequency only within the past ∼5 kya and involving a limited number of large-effect loci (Wilde et al., 2014; Skoglund and Mathieson, 2018).

One of the major pigmentation-associated genes, *SLC24A5,* encodes the potassium-dependent sodium-calcium exchanger NCKX5, which localizes to the trans-Golgi network of melanocytes and plays a key role in melanosomal ion homeostasis and melanogenesis (Lamason et al., 2005). Its derived rs1426654-A allele is now nearly fixed across Europe but remains rare in most African and East Asian populations, consistent with a strong and recent selective sweep dated to approximately 10 kya (Basu Mallick et al., 2013). A similar pattern is observed at *SLC45A2*, which encodes a transporter influencing melanosomal pH and whose derived rs16891982-G allele also reaches high frequency in Europeans, further reducing melanin production (Le et al., 2020). Together, these variants shift melanogenic pathway to reduced melanin production and facilitate sufficient cutaneous vitamin D_3_ synthesis in low UVB exposure, particularly following the transition to vitamin D3-poor agricultural diets (Lucock, 2023; Maitra et al., 2025; Chaplin and Jablonski, 2009).

Although these two loci dominate the European depigmentation signal, additional genes contribute to both baseline pigmentation and its phenotypic variability. The rs1042602 (p.Ser192Tyr) variant of *TYR*, affecting the rate-limiting enzyme in melanin biosynthesis, further reduces melanin levels (Shriver et al., 2003). At the same time, substantial within-Europe diversity arises from epistatic interactions, most notably involving *MC1R* and *ASIP*. *MC1R*, which regulates the balance between eumelanin and pheomelanin synthesis, shows exceptional allelic diversity in Europeans, with several loss-of-function variants (e.g., rs1805008 and rs1805009) associated with fair skin, freckling, and red hair (Makova and Norton, 2005). This unusually high diversity is thought to be maintained by some form of balancing selection (via frequency-dependent selection). *ASIP*, which antagonizes *MC1R* signaling, further modulates this system, although its effect size is modest compared to the major depigmentation loci (Zaorska et al., 2019).

Additional layers of fine-scale pigmentation variation in Europe are contributed by genes such as *IRF4*, *BNC2*, and *UGT1A*. The intronic *IRF4* variant rs12203592 influences skin pigmentation, tanning response, and hair pigmentation, but shows weaker evolutionary signals than the major sweep loci (Praetorius et al., 2013). Variants near *BNC2,* particularly rs12350739, contribute to pigmentation variation through allele-specific regulatory effects (Visser et al., 2015). Besides, genomic surveys of archaic introgression identify Neanderthal-derived haplotypes overlapping this region. It suggests that archaic regulatory variation may still influence the present-day phenotypic diversity (Reilly et al., 2022). *UGT1A*, in contrast, appears to influence pigmentation primarily through metabolic pathways rather than the canonical pathway of melanogenesis. The rs6742078 variant of this gene is strongly associated with quantitative measures of skin hue in Europeans, marking it as a genuine but noncanonical pigmentation locus. However, it shows little evidence of having been a direct target of selection (Jacobs et al., 2013).

Thus, while many loci contribute to fine-scale pigmentation variation in Europe, the continental-scale shift towards depigmentation is primarily driven by a small number of loci with unusually large effects. Polygenic modifiers shape secondary variation rather than the primary phenotypic transition, consistent with a genetic architecture dominated by recent selective sweeps rather than diffuse polygenic adaptation.

#### 2.4.3 South Asia

The selection landscape of skin pigmentation in South Asia reflects overlapping signals of natural selection, ancient admixture, and region-specific environmental and sociocultural pressures. The *SLC24A5* gene has been identified as the principal regulator of skin pigmentation diversity within South Asia, particularly rs1426654, whose derived A allele, almost fixed in Europeans, shows a broad frequency range across South Asian populations and is strongly associated with lighter skin and reduced melanin levels (Stokowski et al., 2007; Basu Mallick et al., 2013; Mishra et al., 2017). Identity-by-descent and phylogeographic analyses indicate that this allele was introduced into South Asia via West Eurasian gene flow and subsequently rose to high frequencies under positive selection in many groups, with its distribution further structured by latitude, ancestry components, and caste-tribe stratification across a heterogeneous UV regime (Basu Mallick et al., 2013; Iliescu et al., 2018; Mukherjee et al., 2013). Interestingly, this variant does not function in isolation, but is embedded within a broad conserved haplotype structure on chromosome 15q21.1, encompassing rs2470102 (Mishra et al., 2017), rs11070627, and rs12913316, located at the adjacent *MYEF2* and *CTXN2* genes, respectively (Sarkar et al., 2018). This genomic cluster, although not all loci within it directly participate in melanogenesis, remains in strong linkage disequilibrium with rs1426654 and shows coordinated segregation in South Asian populations (Sarkar et al., 2018). This pattern reflects a selective sweep centered on *SLC24A5*, with neighboring variants rising in frequency through genetic hitchhiking. This widespread prevalence of the extended *SLC24A5* haplotype and its associated allelic structure across the diverse South Asian groups supports a shared, likely monophyletic origin, consistent with the introduction of the derived rs1426654-A allele (Sarkar et al., 2018). Along with the *SLC24A5*, allelic variants in other pigmentation-related genes such as *SLC45A2* and *TYR*, also affect skin pigmentation diversity within South Asian populations, although their effect sizes are smaller and their frequencies, more spatially and socially structured (Stokowski et al., 2007). Additionally, the intronic variant rs2733832 at *TYRP1* further reveals caste-tribe differences, while Tibeto-Burman–speaking tribal groups in northeastern India show high frequencies of the derived *MC1R* rs885479 (p.Arg163Glu) allele, reflecting their distinct ancestry components and demographic histories (Mukherjee et al., 2013). Recent evidence further identifies rs3212363, a non-coding UTR variant in *MC1R*, which exhibits strong regional stratification within India, indicating an additional population-specific contribution of *MC1R* to pigmentation diversity. The derived T allele is associated with lighter skin pigmentation, with a bias towards male individuals, and is more prevalent in northern and northeastern populations compared to southern Dravidian tribal populations (Kashyap et al., 2026).

Together, these pigmentation-related loci illustrate the polygenic basis of skin pigmentation in South Asia, shaped by heterogeneous selection pressures, ancient admixture layers, and long-standing sociogenetic structuring arising from caste- and tribe-based endogamy (Mukherjee et al., 2013; Kashyap et al., 2026).

#### 2.4.4 East Asia

The evolutionary genetics of skin pigmentation in East Asia illustrates a contrasting pattern in which substantial phenotypic diversity has been orchestrated primarily through the accumulation of numerous moderate- and small-effect variants rather than through the fixation of the major European depigmentation alleles. Many East Asian populations evolved comparatively lighter skin despite retaining the ancestral rs1426654-G allele, demonstrating that convergent depigmentation can emerge through distinct genetic pathways (Norton et al., 2007). This pattern exemplifies a predominantly polygenic mode of adaptation shaped by regionally heterogeneous UV environments and complex demographic histories. Rather than being driven by a few sweeping mutations, East Asian pigmentation variation reflects coordinated shifts across numerous genes involved in melanogenesis and its regulation. Strong selection signals are observed at several region-specific loci, most notably at *OCA2*, *KITLG*, and *SLC24A2*. The rs1800414 (p.His615Arg) and rs74653330 (p.Ala481Thr) variants of *OCA2* modify melanin biogenesis independently of the major European pigmentation alleles (Eaton et al., 2015; Murray et al., 2015). While *KITLG,* encoding the stem cell factor (SCF) that signals through c-KIT receptor, shows strong selection at adaptive loci rs428316 and rs642742 and contributes to lighter pigmentation in low-UV environments at northern latitudes (Yang et al., 2018). *SLC24A2*, another solute carrier involved in ionic homeostasis, carries the derived variant of rs10122939, which influences calcium-cation balance within melanogenic pathways (Wang et al., 2022).

Beyond these major contributors, several GWAS revealed a broad polygenic architecture shaped by additional selected loci, including *ADAM17* (rs4328603), *ADAMTS20* (rs11182091, rs11182085, rs1510523), and *ATRN*, with effect sizes comparable in magnitude to the sweeps observed at *SLC24A5* and *SLC45A2* in Europeans (McEvoy et al., 2006). Other loci, such as *TYRP1* (rs10809814) and *DCT* (rs1407995), further reinforce the polygenic basis of East Asian pigmentation (Del Bino et al., 2018). Several non-canonical genes, including *EGFR* and *DRD2*, have also contributed to the regional pigmentation differences, highlighting the role of keratinocyte-melanocyte crosstalk and pleiotropic regulatory influences on pigmentation pathways (Lao et al., 2007). This polygenic structure is further supported by a recent large-scale GWAS that identified twenty-three loci, including eleven novel variants mapping to or near the genes such as *USP35*, *BCKDHB*, *GAB2*, and *MRPS22*, and estimated the SNP-based heritability at ∼23–24%. These results demonstrated that East Asia-specific pigmentation loci diverge substantially from those identified in Europeans, consistent with signatures of largely independent polygenic adaptation (Kim et al., 2024). However, convergent signals at *ASIP* and *BNC2* suggest the retention of some shared Eurasian components predating the divergence of European and East Asian lineages (Del Bino et al., 2018).

Local ecological extremes have further layered additional adaptive modifiers onto this polygenic background. In Tibetans, strong selection targets the intronic enhancer variant rs75356281 in *GNPAT*, which shows UV-inducible enhancer activity. Functional assays demonstrate that the derived rs75356281-T allele increases *GNPAT* expression and enhances UV-induced melanogenesis, thereby improving UV protection at high altitude (Yang et al., 2022). Conversely, in regions of East Asia with low UVR, such as among Han Chinese, the derived T allele of the *PAH* rs10778203 variant decreases UV-induced enhancer activity and weakens tanning responsiveness, contributing to region-specific lightening through attenuated facultative pigmentation (Pu et al., 2024). At the extreme north of East Asia, indigenous Siberian populations such as Eskimos, Chukchis, and Koryaks exhibit a high frequency (∼64%) of the rs4264393-G variant of *PRDM7*, encoding a methyltransferase, with regulatory contribution to melanocyte gene activity (Malyarchuk, 2022). Paleogenomic evidence traces this variant back to ∼10 kya among ancient Siberian populations during Late Pleistocene migrations, reflecting an ancient region-specific Northeast Asian signal with potential regulatory effects on pigmentation (Malyarchuk, 2022).

Together, these findings indicate that depigmentation in East Asia reflects a largely independent and strongly polygenic evolutionary trajectory, demonstrating that broadly similar phenotypes can arise through fundamentally different genetic architectures.

#### 2.4.5 Native America

The genetic structure of skin pigmentation in Native American Populations reflects a complex combination of inherited East Asian ancestry, subsequent demographic history, and region-specific modification across heterogeneous UV environments. Phenotypic diversity, therefore, arises largely from polygenic variation operating within a distinctive Indigenous genomic background. Unlike populations in Africa and Europe, their phenotypic diversity cannot be attributed solely to the major hypopigmentation alleles found in Europeans or the high melanin content typical of African populations, suggesting the presence of lineage-specific pigmentation-modifying variants (Adhikari et al., 2019; Ang et al., 2023).

Since Native Americans share ancestry with East Asians, several light-pigmentation alleles, particularly those that reached fixation in their common ancestors, likely predate the southward migration from Beringia (Ang et al., 2023). However, classical European depigmentation alleles, particularly *SLC24A5* rs1426654-A and *SLC45A2* rs16891982-G, contribute to lighter pigmentation mainly in admixed groups, but their phenotypic effect sizes are often attenuated by Native American genomic background and epistatic interactions with other loci (Deng and Xu, 2018). This pattern is illustrated in the Caribbean populations such as Kalinago of Dominica, who retain >50% Native American ancestry yet exhibit darker pigmentation than predicted by known European hypopigmentation variants, implying the presence of additional, as-yet-unidentified Native American-specific variants (Quillen et al., 2019; Ang et al., 2023).

Large-scale analyses from the CANDELA consortium (Adhikari et al., 2019) further reveal that pigmentation in Latin Americans is highly heritable and shaped by tri-hybrid ancestry (Native American, European, African). Associations consistently localize to canonical pigmentation loci such as *SLC24A5*, *SLC45A2*, *OCA2/HERC2*, and *GRM5/TYR*, but their effect sizes vary substantially depending on the local genomic background. Novel variants at *MFSD12*, particularly rs2240751 (p.Tyr182His), are enriched in Native American and East Asian populations but largely absent in Europeans, suggesting the convergent evolution for depigmentation through different genetic routes under a similar pattern of lower solar radiation (Adhikari et al., 2019). Pleiotropic loci at non-canonical pigmentation genes, such as the *EGFR* and the *OPRM1,* further indicate that pigmentation differences extend beyond core melanogenic networks and may involve skin-barrier and keratinocyte or broader stress-response pathways (Jablonski, 2021; Quillen et al., 2012).

Notably, much of the current understanding of pigmentation genetics in the Americas derives from the admixed populations, including Afro-Americans, Cubans, Brazilians, Puerto-Rican cohorts, where classical European pigmentation-associated loci such as *SLC24A5* and *SLC45A2* consistently emerge as major determinants of pigmentation variation (Marcheco-Teruel et al., 2014; Lona-Durazo et al., 2019; Durso et al., 2014; Hernandez-Pacheco et al., 2017). In contrast, large-scale genome-wide studies specifically conducted in indigenous American populations remain comparatively scarce. Consequently, the extent to which currently identified loci contribute to the Native American-specific pigmentation variation is likely uncertain.

The skin pigmentation of Native Americans does not consistently align with the prediction of the classic Vitamin D-Folate hypothesis (Missaggia et al., 2020). Though environmental adaptation has contributed, the patterns appear more complex than in Eurasia. In some higher-latitude regions, lighter pigmentation may facilitate vitamin D_3_ synthesis, but dietary practices, behavioural adaptation, and demographic history likely buffered UV-related constraints, weakening simple clinal relationships between latitude and skin pigmentation (Lucock, 2023; Milks and Brown, 2024). The skin pigmentation of Native Americans is best understood as a distinct, ancestry-modulated, highly polygenic system shaped by regionally variable selective pressures and extensive admixture. This diversity underlines the polygenic and context-dependent nature of pigmentation evolution in the Americas and reveals that a substantial portion of human adaptive diversity is still genetically unexplored, particularly in historically underrepresented populations.

#### 2.4.6 Southeast Asia and Oceania

The diversity of skin pigmentation observed among indigenous populations of Southeast Asia and Oceania reflects the intricate interplay between deep ancestral continuity, genetic inheritance, local environmental adaptation, and prolonged demographic isolation. Archaeological and genomic evidence indicates that early modern human dispersals through southern Asia and Island Southeast Asia reached Sahul approximately 50–70 kya, contributing to the ancestry of present-day Papuan, Melanesian, and Aboriginal Australian populations (Bae et al., 2017). Several Indigenous groups of Peninsular Malaysia and the Philippines, historically referred to in parts of the literature as ‘Negrito’, also retain deeply divergent ancestry components related to early population structure in Asia and Sundaland. However, their genetic histories are heterogeneous and reflect subsequent drift, isolation, and admixture with later East Asian-, Austroasiatic-, and Austronesian-related populations (Aghakhanian et al., 2015; Larena et al., 2021). Across these geographically dispersed populations, deeply melanized phenotypes are common and are consistent with long-term exposure to tropical high-UV environments. Nevertheless, the evolutionary pathways underlying these phenotypes likely involve both ancestry-mediated inheritance and population-specific or convergent adaptive processes.

Recent comparative genomic analyses have identified several candidate pigmentation-associated loci in these populations. Adaptive alleles at *OCA2* reported in Philippine Indigenous groups (rs2703923-G) and in Papuan populations (rs28003335-A) overlap with alleles prevalent in broader Asian populations and may partly reflect shared ancestry or recent admixture. Additional population-specific signals have been reported at *MEGF11* and *IRF4* in some Malaysian Indigenous groups (‘Negritos’), *RNF111* and *SLFN12L* in Philippine Indigenous groups, and *TUSC3*, *LINC00504*, and *PLEKHA6* in Papuan populations (Deng et al., 2022). These findings suggest that pigmentation evolution in Southeast Asia and Oceania has been shaped by both shared ancestral variation and regionally differentiated adaptive histories. However, compared with these emerging signals of local adaptation, the genetic basis of skin pigmentation remains underexamined across much of Southeast Asia and Oceania. This limitation is particularly evident among the Melanesian population, where exceptionally dark pigmentation is well documented, but only a few pigmentation-associated variants have been robustly characterized among them. Melanesians, particularly those inhabiting Island Melanesia, possess some of the darkest skin pigmentation globally, with individuals from Bougainville displaying the highest melanin indices observed in any living human group (Jablonski and Chaplin, 2000; Norton et al., 2006). Historical accounts further describe comparably profound pigmentation among Aboriginal Tasmanians (Diamond, 2005). This extreme pigmentation is not solely the product of shared ancestral alleles with African populations but also reflects unique evolutionary trajectories, including the retention of ancient variants and the emergence of region-specific adaptations such as *TYRP1* (Kenny et al., 2012). Its rs387907171-T variant (p.Arg93Cys), which has been widely identified as Melanesian blonde hair variant, also demonstrates a modest explanatory power (∼5.3%) to the variance in skin pigmentation (Kenny et al., 2012).

Melanesians also exhibit distinctive genetic architectures of pigmentation. In particular, *MC1R* shows high diversity but no evidence of functional association with pigmentation levels, nor signals of strong purifying selection, distinguishing Melanesians from Europeans, where *MC1R* variations have major phenotypic effects (Norton et al., 2015). These findings suggest that pigmentation in Melanesia is likely governed by genetic mechanisms independent of *MC1R*; however, the Melanesia-specific studies do not identify the causal loci responsible. Although research in other global populations has highlighted additional eumelanin-regulating pathways, such as *MFSD12* and the *DDB1/TMEM138* regulatory region, these genes have not yet been functionally evaluated for their contributions to Melanesian pigmentation.

Aboriginal Australian populations exhibit similarly dark pigmentation, widely interpreted as a long-standing adaptation to the intense UV radiation characteristic of the Australian continent (Norton et al., 2015). Ancient migration routes, prolonged geographic isolation, and minimal admixture likely preserved these distinctive pigmentation profiles. This dual mechanism of ancestral retention and convergent evolution, reinforced by traditional lifeways such as minimal clothing and vitamin D3-rich diets, has led to deeply eumelanin-rich skin pigmentation, beyond which further darkening is neither observed nor advantageous (Chaplin and Jablonski, 2009). Together, the pigmentation patterns of indigenous populations of Southeast Asia and Oceania exemplify independent but parallel histories of strong UV-driven selection, in which deeply eumelanin-rich skin evolved and persisted without reliance on the depigmentation pathways that characterize Eurasian populations. Nevertheless, compared with African, European, and East Asian populations, the genetic architecture of skin pigmentation in these populations remains incompletely characterized, with many candidate loci yet to undergo functional validation. This emphasizes a major priority of future pigmentation genomic studies in these historically underrepresented populations.

At a broad theoretical level, the diverse regional patterns reviewed above can be understood as different combinations of a few recurring evolutionary processes: (i) hard-sweep adaptation driven by large-effect variants, (ii) diffuse polygenic adaptation involving many small-to-moderate effect variants, (iii) long-term stabilizing selection and purifying selection around a deeply melanized ancestral state, and (iv) reshaping of genetic architectures through admixture. Rather than fitting neatly in one category, continental populations reflect a distinct combination of these processes, shaped by their particular demographic and environmental history (see Table 1 for comparative summary).

**TABLE 1:** Comparative summary of evolutionary architecture of human skin pigmentation across populations.

Region	Ecological context	Main evolutionary process	Key pigmentation genes/loci	Key variants	Demographic modifiers	Main interpretive point
Africa	Persistent high UVR; regional ecological heterogeneity	Long-term stabilizing selection for dark pigmentation; local adaptation	*MFSD12*, *DDB1*/*TMEM138*, *PDPK1*	*MFSD12* rs6510760	Regional population structure; reverse migration; pastoralist gene flow	Diversity reflects fine-scale demographic history rather than simple UV cline
Europe	Low UVB; post-glacial environments; agricultural transition	Strong recent directional selection (hard sweeps) on large-effect loci	*SLC24A5*, *SLC45A2*, *TYR*, *MC1R*, *ASIP*, *IRF4*, *BNC2*	*SLC24A5* rs1426654; *SLC45A2* rs16891982	Ancient admixture of Neolithic farmers and Steppe migrations	Depigmentation largely driven by few large-effect variants
South Asia	Intermediate, heterogeneous UVR; latitudinal gradient and sociocultural stratification	Adaptive introgression with selective sweep; Polygenic effects	*SLC24A5*, *SLC45A2*, *TYR*, *TYRP1, MC1R*	*SLC24A5* rs1426654	West Eurasian admixture; caste- and tribe-based endogamy; population structure shaped by ancient components	Admixture-driven selective sweep embedded in complex socio-genetic framework
East Asia	Variable UVR; northern low UV; high-altitude extremes	Predominantly polygenic adaptation; convergent evolution; regional selection	*OCA2*, *KITLG*, *SLC24A2*, *GNPAT*, *PAH*, *PRDM7*	*OCA2* rs1800414	Complex demographic history; regional ecological adaptation	Convergent depigmentation via independent polygenic pathways
Native America	Broad UV gradient; Arctic to tropical; dietary buffering	Polygenic adaptation; ancestry-specific modifiers; admixture effects	*SLC24A5*, *SLC45A2*, *OCA2/HERC2*, *MFSD12*, *EGFR*	*MFSD12* rs2240751, *OCA2* rs1800404	Tri-hybrid admixture (Native American, European, African); Beringian ancestry	Ancestry-modulated and context-dependent pigmentation, insufficient classic UV models
Southeast Asia and Oceania	High UV exposure; island isolation	Strong stabilizing selection for dark pigmentation; ancestral retention	*TYRP1*	*TYRP1* rs387907171	Deep isolation; minimal admixture; ancient migration routes	Deep pigmentation evolved independently

### 2.5 Gene-culture feedback loop and the evolutionary trajectory of human skin pigmentation

The genetic architecture and evolutionary trajectories described above did not occur in isolation, but were embedded within ecological and cultural contexts that continually modify the extent and intensity of UVR exposure. Increasingly, research emphasizes that global variation in human skin pigmentation cannot be explained by biological selection alone, but instead reflects a complex biocultural phenomenon (Jablonski, 2021). Indeed, human skin pigmentation is often cited as a compelling example of gene-culture co-evolution (GCC), in which genetic adaptations and cultural innovations interact reciprocally over a long evolutionary timescale (Kasser et al., 2025). Cultural behaviors, including clothing use, shelter construction, changes in subsistence strategies, dietary practices, and migration altered the UVR-related exposome experienced by different populations. Diets rich in vitamin D3 (e.g., marine-based foraging, terrestrial game hunting, sun-dried food), along with symbolic and cosmetic practices such as body painting, and technological innovations in garment production, served as cultural buffers that could either soften or intensify UVR-related constraints (summarized in Table 2). At the genetic level, the culturally mediated exposome did not simply influence biological selection but modified its parameters. By altering effective UVR exposure, cultural practices modify the strength and even direction of selection acting on pigmentation loci, and shift adaptive optima. Thus, gene-culture feedback helps to explain why some contemporary populations maintain dark pigmentation at high latitudes, why depigmentation has followed different genetic routes in different regions, and how cultural practices operating across different timescales, from long-standing hunter-gatherer lifeways to very recent agricultural transitions, have interacted with admixture and standing genetic variation. These factors often play a larger role than simple selective sweeps.

**TABLE 2:** Cultural factors and their evolutionary consequences on skin pigmentation.

Cultural factor	Effect on selection regime	Empirical examples
Migration	Creates mismatch between inherited pigmentation levels and local UVR regime, altering the tempo and direction of microevolution at pigmentation loci	Migration of Bantu population to southern Africa (∼1,500–500 years ago), who retain dark pigmentation adapted to their original habitat of equatorial regions, in contrast to lighter-skinned KhoeSan populations, shaped under lower UVR exposure (Lin et al., 2018)
Clothing	Reduces effective UVR exposure in intense UV regime; thereby buffering UV-related trade-off and modifying the strength of selection on pigmentation genes	Petroglyph evidence from mid-Holocene Arabia (∼5 kya) depicts body-covering elongated garment with head-coverings (Baumer, 2022), a clothing tradition that persists among many Arabian populations today, contrasted with the much longer inhabitants, Nuer and Dinka on the western side, who retain dark pigmentation (Jablonski and Chaplin, 2002)
Subsistence shift (e.g., Neolithic revolution)	Alters pigmentation selection dynamics because of the transition from vitamin D3-laden animal-based hunting to cereal-based agriculture	aDNA evidence indicates a rapid increase in depigmentation-associated alleles following the Neolithic transition (Mathieson et al., 2015; Irving-Pease et al., 2024), consistent with the hypothesis that agriculture intensified selection for lighter pigmentation in low-UVR latitudes to compensate for reduced dietary vitamin D3 intake (Lucock, 2023), although the extent to which these selective shifts reflect dietary change, rather than demographic processes or standing genetic variation, remains unresolved
Diet (Vitamin D3 intake)	Weakens selection for depigmentation genes, by reducing reliance on cutaneous vitamin D3 synthesis	Inuit population (aquatic hunter-gatherers) at extreme northern latitudes and the Sami population of Finland, whose traditional diets are rich in vitamin D3 from marine resources (Juzeniene et al., 2009) and reindeer meat, respectively, experience less selective pressure for depigmentation
Symbolic practices (e.g., body painting or applying topical agents on the body)	Provides partial photoprotection, mimicking the function of melanin; potentially decreases selection for dark skin pigmentation	Archaeological evidence for early use of red ochre mixed with animal fat, as a topical photoprotective agent, as found in African populations (Rifkin et al., 2015)

## 3 Conclusions and future directions

Human skin pigmentation represents a compelling example of adaptive evolution. Far from being a static trait, it reflects a dynamic evolutionary narrative, embedded in global patterns of migration, selection, sociocultural, and behavioral adaptation. The evidence synthesized here in this review demonstrates that UVR has been a major selective force underlying global pigmentation diversity, yet no single evolutionary model sufficiently explains the observed patterns. Instead, pigmentation evolution reflects a multilayered adaptive landscape in which UVR-mediated selection interacts with physiological constraints, demographic history, and sociocultural buffering. Advances in genomics further demonstrate that similar pigmentation phenotypes have evolved through distinct genetic routes across populations, ranging from large-effect selective sweeps in Europe to predominantly polygenic adaptation in East Asia, ancestry-specific architectures in Africa, South Asia, and regionally variable selective pressures in the Americas, Southeast Asia, and Oceania. Ancient DNA studies further demonstrate that many present-day pigmentation patterns are evolutionarily recent and have been strongly influenced by migration, admixture, and changing ecological conditions. Taken together, these findings support a view of human skin pigmentation not as a simple latitudinal response to UVR gradient, but as a dynamic biocultural trait whose evolutionary trajectory reflects the interaction of natural selection, population history, and gene-culture coevolution. It accentuates the need of a more integrative perspective that combines functional genomics, sociogenomics, environmental modeling, and population history.

Although substantial progress has been made in elucidating the adaptive significance and genetic architecture of human skin pigmentation, important research gaps remain regarding functional consequences of newly identified loci, the representation of globally diverse populations, and the temporal dynamics through which pigmentation-associated variants emerged and spread. Addressing these challenges, several research priorities have emerged:

### 3.1 Functional validation of emerging genes

Recent genomic studies (Bajpai et al., 2023; Martin et al., 2017) have identified several novel and non-canonical pigmentation genes, such as *KLF6*, *COMMD3*, *SNX13*, *SMARCA2*, *ADAM17*, *ADAMTS20*, that extend beyond the classical melanogenic pathways. These candidate genes require experimental validation to understand their functional roles to move from statistical association to biological inference. Future research should integrate CRISPR-Cas9-mediated gene editing in primary human melanocytes with 3D organotypic skin models, such as Human Epidermal Equivalents (HEEs) (Yoon et al., 2003; Hall et al., 2022; De Los Santos Gomez et al., 2026), coupled with immunohistochemistry and transcriptomic profiling. These approaches will enable systematic analysis of melanocyte-keratinocyte interactions, subcellular trafficking processes, and regulatory networks that drive pigmentation diversity.

### 3.2 Expanding genomic diversity in research

Despite major progress, pigmentation genomics remains disproportionately focused on European populations. Expanding representation to include different African, South Asian, Southeast Asian, Indigenous American, and Australo-Melanesian populations, including endogamous, tribal, and hunter-gatherer groups, is essential for reconstructing the entire evolutionary landscape of human pigmentation.

Multi-ethnic genomic consortia with a large-scale and robust framework can facilitate rigorous analyses of gene-gene and gene-environmental interactions. It can unravel the dynamics of the human genome, including the ancestry patterns, region-specific adaptive responses, and convergent evolutionary trajectories, which can further enhance precision genomic medicine across varied human populations.

### 3.3 Leveraging recently admixed populations

Populations with recent and substantial admixture provide the framework to investigate how pigmentation-associated alleles interact with diverse genomic and ecological contexts. Advances in admixture mapping, high-throughput phenotyping, and environmental exposure profiling can further decipher the interplay among ancestry, adaptive selection, and phenotypic plasticity in shaping contemporary pigmentation diversity.

### 3.4 Integrating ancient DNA (aDNA)

Augmenting research on diverse extant indigenous and admixed populations with aDNA data introduces crucial chronological perspectives to the study of pigmentation evolution. Time-transects of ancient genomic data from diverse regions, specifically from Africa, South Asia, East Asia, and Oceania, are essential in predictive modeling of the spatiotemporal movement of alleles as they migrate, introgress, and adapt in shifting environmental pressures and evolving socio-cultural frameworks. New statistical methodologies for detecting selection, epistasis, and adaptive introgression in temporal data with paleoenvironmental reconstructions and archaeogenomic evidence will allow for increasingly precise modeling of the spatiotemporal dynamics of pigmentation evolution.

### 3.5 Synthesis

Together, these research directions indicate a paradigm shift in the study of human skin pigmentation, extending beyond cataloguing variants and toward a more comprehensive, mechanistic understanding of pigmentation biology. The convergence of high-throughput multi-omics, advanced functional models, broader population representation, and unified analyses of ancient and modern genomes provide opportunity to reconstruct the evolutionary narrative of skin pigmentation with unprecedented resolution.

Future research that synthesizes functional, ecological, and sociogenomic determinants, and that considers ethical inclusivity and methodological rigor, can extend the field beyond a largely descriptive study into a translational framework. Such a framework will not only elucidate the evolutionary basis of pigmentation but can also enhance our understanding of human adaptation, genomic diversity across populations, and the equitable application of biomedical advances.
